# A biomimetic gelatin-based platform elicits a pro-differentiation effect on podocytes through mechanotransduction

**DOI:** 10.1038/srep43934

**Published:** 2017-03-06

**Authors:** Mufeng Hu, Evren U. Azeloglu, Amit Ron, Khanh-Hoa Tran-Ba, Rhodora C. Calizo, Iman Tavassoly, Smiti Bhattacharya, Gomathi Jayaraman, Yibang Chen, Vera Rabinovich, Ravi Iyengar, James C. Hone, John C. He, Laura J. Kaufman

**Affiliations:** 1 Department of Mechanical Engineering Columbia University, New York, NY 10027, USA; 2 Department of Pharmacological Sciences and Systems Biology Center New York, Icahn School of Medicine at Mount Sinai, New York, NY 10029, USA; 3 Department of Chemistry Columbia University, New York, NY 10027, USA; 4 Division of Nephrology, Department of Medicine, Icahn School of Medicine at Mount Sinai, New York, NY 10029, USA

## Abstract

Using a gelatin microbial transglutaminase (gelatin-mTG) cell culture platform tuned to exhibit stiffness spanning that of healthy and diseased glomeruli, we demonstrate that kidney podocytes show marked stiffness sensitivity. Podocyte-specific markers that are critical in the formation of the renal filtration barrier are found to be regulated in association with stiffness-mediated cellular behaviors. While podocytes typically de-differentiate in culture and show diminished physiological function in nephropathies characterized by altered tissue stiffness, we show that gelatin-mTG substrates with Young’s modulus near that of healthy glomeruli elicit a pro-differentiation and maturation response in podocytes better than substrates either softer or stiffer. The pro-differentiation phenotype is characterized by upregulation of gene and protein expression associated with podocyte function, which is observed for podocytes cultured on gelatin-mTG gels of physiological stiffness independent of extracellular matrix coating type and density. Signaling pathways involved in stiffness-mediated podocyte behaviors are identified, revealing the interdependence of podocyte mechanotransduction and maintenance of their physiological function. This study also highlights the utility of the gelatin-mTG platform as an *in vitro* system with tunable stiffness over a range relevant for recapitulating mechanical properties of soft tissues, suggesting its potential impact on a wide range of research in cellular biophysics.

Kidney podocytes are adhesion-dependent differentiated epithelial cells in the renal corpuscle that perform a critical filtration function in urine formation. Healthy podocytes reside on a layer of glomerular basement membrane (GBM) with interdigitating foot processes connected by a nanoporous protein network, called the slit diaphragm, that acts as a filter. In several progressive nephropathies, including glomerulosclerosis, alterations in GBM composition, physical structure, and mechanical properties occur. These changes are accompanied by effacement of podocyte foot processes and deterioration of podocyte function^1^,^2^,^3^,^4^,^5^,^6^,^7^. These observations suggest that the physical properties of GBM are important for podocytes to maintain a differentiated phenotype in tissues, and mechanical changes to GBM may result in progressive renal dysfunction. While it is well known that tissue stiffness is a critical cue guiding cell growth and differentiation^8^,^9^,^10^,^11^,^12^, whether and how podocytes respond to changes in matrix stiffness has not previously been explored. Here, we hypothesized that podocyte sensing of substrate stiffness could drive podocyte differentiation and biochemical specialization and that optimal phenotypic response would occur at glomerular physiological stiffness. In order to test this hypothesis – and given that podocytes fail to form foot processes with slit diaphragms when cultured on standard substrates – we employed a biomimetic based hydrogel culturing system that allows tuning stiffness independent of chemical composition. This allowed analysis of the effect of substrate stiffness on podocyte phenotype and differentiation and functional markers. The substrates used were composed of pharmaceutical grade gelatin (hydrolyzed collagen) crosslinked by a natural microbial transglutaminase (mTG) enzyme (Fig. 1). These substrates, in contrast to un-crosslinked gelatin, are solid at standard cell culture temperature and can be prepared with a range of stiffness overlapping those of tissues including healthy glomeruli. Figure 1 shows that varying mTG reactivity from 0.6 U to 30 U increased the Young’s modulus of the gelatin from 0.6 kPa to 13 kPa, providing substrates with a range of stiffness encompassing that reported for healthy and diseased glomeruli. Using a variety of approaches, intact healthy glomerulus has been measured as having Young’s modulus of ~2.5–4.0 kPa, with diseased glomerulus demonstrating stiffness both lower (Alport disease and HIV-associated nephropathy models) and higher (fibrotic disease models)^13^,^14^,^15^.

Preparation of gelatin-mTG substrates requires only straightforward mixing of gelatin and mTG solutions. This simple process increases fabrication efficiency and helps to limit gel to gel variability compared with other crosslinked gelatin systems, and the only obvious limitation of the gelatin-mTG system relative to other crosslinked gelatin systems is that it does not have a porous structure, potentially limiting its utility for 3-D cell culture^16^,^17^,^18^,^19^,^20^,^21^,^22^. The gelatin-mTG system also has several advantages over commonly used stiffness-tunable synthetic materials such as polyacrylamide (PAA) gels and polydimethylsiloxane (PDMS) elastomers. One key advantage for the study of kidney podocytes relates to the ability to create large, homogeneous culture areas with the gelatin-mTG system. Podocytes in culture exhibit relatively low functional biomarker expression^23^, and it is therefore necessary to culture a large number of podocytes in each condition of interest to make assays feasible. Additional and related advantages of the gelatin-mTG system include that it is inexpensive to prepare, toxin-free, and it does not require coating with ECM proteins. While PAA gels require Sulfo-SANPAH to serve as an ECM linker, potentially leading to sub-optimal ECM coating, mTG-crosslinked gelatin contains RGD tripeptides, allowing for cell adhesion even without ECM coating^24^,^25^. Moreover, whereas crosslinkers and unpolymerized acrylamide are toxic to cells, gelatin crosslinked by mTG produces only ammonia as a byproduct, which is straightforward to remove. Poly(dimethyl siloxane) elastomers, which are also commonly used for culture on soft substrates, also require additional ECM coating^26^,^27^ and cannot generally be prepared with stiffness below ~5 kPa, higher than that of intact renal glomerular tissue^13^,^14^,^15^.

## Materials and Methods

### Preparation of Transglutaminase Crosslinked Gelatin with Varying Stiffness

The gelatin stock solution was prepared at a fixed concentration of 10% w/v. Gelatin (Type A from porcine skin, 300 bloom, Sigma) powder was heated and solubilized in 1x Dulbecco’s phosphate-buffered saline (DPBS) at 50 °C. The solution was transferred to a humidified chamber at 37 °C and held there for at least 30 min until the solution cooled to 37 °C. Dissolution of 0.006 g, 0.01 g, 0.03 g and 0.3 g of mTG (Activa-TI, Ajinomoto Inc., activity of 100 U per gram of mTG, per gram of protein according to the manufacturer) was performed in 1x DPBS at 37 °C to give 0.6 U, 1 U, 3 U, and 30 U reactivity, respectively. To prepare each hydrogel sample, a 7 mL gelatin and a 3 mL mTG solution were well mixed, and the final solution was incubated at 37 °C for 5 h for crosslinking. Next, gelatin-mTG gels were transferred to an oven at 55 °C for 30 min to deactivate the enzyme. Gels were then soaked in 10 mL of 1x DPBS for 24 h at 37 °C. This gave the target elastic moduli of 0.6  kPa, 2 kPa, 5 kPa and 13 kPa as measured by rheology. In this study of human podocyte mechanotransduction, we used four different ECM coatings: collagen I (CB-40235 Corning™) coating was primarily used, while other ECM coatings – collagen IV (CB-40245 Corning™), fibronectin (CB-40008A Corning™), and laminin (CB-40232 Corning™) – were utilized to verify cellular responses. In all cases, coating at 5 μg/ml (collagen I) or 1 μg/ml (collagen I, collagen IV, fibronectin, and laminin) was performed as follows: following crosslinking with mTG and soaking to remove ammonia, diluted stock ECM solution was pipetted atop the gels. The gels were then incubated at room temperature for one hour and remaining solution was carefully aspirated. For gels with collagen I or IV coating, gels were additionally rinsed with DPBS to remove the acid in the ECM stock solution.

### Rheology of Gelatin-mTG Gels

The characterization of gel mechanical properties was performed on an Anton Paar MCR 302 WESP rheometer with a built-in temperature and gap calibration. A metal parallel plate geometry (Anton Paar PP25, 25 mm in diameter) was utilized in oscillation mode. A strain sweep measurement was first conducted with strain amplitude γ = 0.1%–100% and fixed frequency *f* = 1 Hz to determine the linear regime of γ (Figure S1). The frequency was then swept from 0.1–10 Hz (5 data points per decade) at γ = 1% (Figure S1) and storage and loss moduli were recorded at 1 Hz. The gelatin gels were prepared in 60 mm x 15 mm culture dishes and mounted on the Peltier element (Anton Paar, P-PTD200/GL) with double-side tape to avoid slippage. All gels were kept in an incubator at 37 °C for 1 day and were quickly transferred to the rheometer to prevent gel cooling. All rheology measurements were performed at 37 °C. The gap size for each gel was determined as the gap at which a force of 0.5 N was measured. Due to the variation in the enzyme activity and gel thickness, the gap size varied between 0.54–1.86 mm. All measurements were repeated six times with the exception of the strain sweep, which was done once for each condition. The conversion of shear modulus to Young’s modulus is governed by:


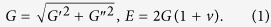


*G* is the shear modulus, which in complex form is expressed as 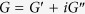. 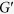 is the shear storage modulus and 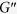 is the shear loss modulus. *v* is the Poisson ratio and *E* is the Young’s modulus. Here, a Poisson ratio of 0.5 was used as suggested by a previous study^28^.

### Cell Culture

Human podocytes were a kind gift from Prof. Moin Saleem (Faculty of Medicine and Dentistry, University of Bristol, UK). The conditionally immortalized human podocytes^29^ were plated on flasks and proliferated at 33 °C for 7 days with RPMI medium (Invitrogen, Cat: 11875119), supplemented with 10% fetal bovine serum (Invitrogen Cat: 25140-079), 1% insulin/transferrin/selenium liquid media supplement (Sigma-Aldrich, Cat: I3145), and 100 units/ml penicillin (Invitrogen, Cat: 15140-122). Cells were then stored at −80 °C for future use. Before each experiment, cells were recovered and cultured in the medium described above at 37 °C in a humidified incubator containing 5% CO_2_ for five days. With an expected confluence of 50% after five days, cells were collected with trypsin EDTA (Invitrogen, Cat: 25300054) and re-plated on different substrates at a density of 2 × 10^5^ cells/100-mm dish.

### Cell Staining

Gel thickness for stained samples was kept below 0.6 mm (e.g. 300 μl gel mixture in a 35 mm culture dish) to avoid optical scattering by the gel. Cells were fixed after 5 days of culture with 4% paraformaldehyde in PBS for 20 min at room temperature. Fixed cells were permeabilized with 0.5% Triton X-100 in PBS for 10 min at room temperature, gently washed and blocked in 4% bovine serum albumin and incubated with primary antibodies for nephrin (Enzo, Cat: 810-016-R100) and podocin (Sigma, Cat: P0372) overnight at 4 °C. Then cells were washed and incubated with anti-rabbit (Alexa Fluor 488, Cell Signaling, Cat: 4412 S) or anti-mouse (Alexa Fluor 547, Cell Signaling, Cat: 4410 S) secondary antibodies for 2 hours. To define cellular boundaries and nuclei, phalloidin (Alexa Fluor 568, Invitrogen, Cat: A12380) and Hoechst 33342 (Invitrogen, Cat: H3570) were applied for 15 and 20 minutes, respectively. A Zeiss LSM710 laser scanning microscope equipped with software platform ZEN Lite 2011 was used to acquire images under identical conditions (gain, pinhole, digital offset). Stack imaging of phalloidin stained cells was performed to acquire at least 30 images spaced 0.33 μm apart, which covered the full cellular profile.

### Motility Assay and Morphological Analysis

Thin layers (~1 mm) of gelatin gels were made to cover the culturing surface of 60 mm x 15 mm culture dishes. Cells were plated on gels with different stiffness and cultured at 37 °C in a humidified incubator containing 5% CO_2_ for 3 days. Phase contrast live cell images were taken in an imaging incubation system (VivaView; Olympus, Japan) every 10 minutes for 48 h and analyzed using ImageJ. From these images, 25 serial images separated by 100 min were selected. Average displacement of each cell was measured by comparing the locations of the cell shape centroid at each time point. Cell motilities were then derived by dividing the average displacement by the elapsed time, and these are expressed in μm/min. Cell morphologies were represented by average cell contour in these 25 images. Spread areas were calculated based on the contour of the cell, while the aspect ratio was based on a fitted ellipse representing the ratio of the long axis to the short axis.

### Cell Proliferation and Viability Assay

Cells were plated on a 96-well plate (500 to 800 cells per well) in media. Prior to cell plating, gelatin gel was crosslinked inside the wells to cover the bottom area. Cells were cultured for 5 days before performing an MTT (3-(4,5-dimethylthiazol-2-yl)-2,5-diphenyltetrazolium bromide) assay. 20 μl of MTT was added to each well containing 100 μl podocyte culturing media. Luminescent signals from the MTT assay (CellTiter 96@ AQueous, Promega Corporation, USA) were read after 4 h. Cell proliferation and viability was calculated by normalizing each luminescent reading from wells with cells on gels over wells without cells on gels. The normalized data were then normalized again over control. Assays were run in triplicate.

### Collection of Cell Lysates and Western Blot Analysis

Cells were collected and lysed in RIPA buffer containing 50 mM Tris-HCl (pH = 7.4), 150 mM NaCl, 0.1% SDS, 0.6% sodium deoxycholate, 1 mM sodium orthovanadate, and 1% Nonidet P-40. Halt protease and phosphatase inhibitor cocktail was added at a 1:50 volume ratio (Thermo Fisher Scientific Cat: 78443) into the buffer. After measurement of protein concentration using a standard Bradford assay, lysate was supplemented with SDS-Sample buffer 4X (Boston BioProducts Cat: BP-110R) at a 1:3 volume ratio and boiled at 95 °C for 5 minutes. 50–80 μg of total proteins were electrophoretically transferred onto nitrocellulose paper. After blocking with casein blocking buffer for two hours (Rockland Inc, Cat: MB-070), blots were incubated with specific primary antibodies against nephrin (Novus Biologicals, Cat: NBP1-30130), WT-1 (Novus Biologicals, Cat: NB120-15249), podocin (Sigma-Aldrich, Cat: P0372), CD2AP (Cell Signaling, Cat: 2135), synaptopodin (Progen, Cat: 55294), phospho-Y416-Src (Cell Signaling, Cat: 6943), phospho-T423-PAK (Cell Signaling, Cat: 2601), GAPDH (Sigma-Aldrich, Cat: G8795), and α-tubulin (Sigma-Aldrich, Cat: T5158) at 4 °C overnight. Then, blots were incubated with horseradish peroxidase-conjugated secondary antibodies (Thermo Fisher Scientific, Cat: 31430, Cat: 31450) at room temperature. Immunocomplexes were visualized subsequently by fluorography using chemiluminescence detection reagent (Thermo Fisher Scientific, Cat: 1859701, Cat: 1859598). Size standards were used to confirm molecular weights (Thermo Fisher Scientific, Cat: 26616). Quantitative comparison of blot intensity was performed using ImageJ software. The band intensities on gels and control of any marker were first converted to percentages of a total intensity and were then normalized by α-tubulin. Final values of relative ratios were rendered by comparing to control.

### RNA Isolation and qRT-PCR

Total RNA was obtained using RNeasy kit (Qiagen, Cat: 74104) according to manufacturer’s instructions. For RT-PCR, first-strand cDNA was prepared from total RNA (~2ug per reaction) using SuperScript III first strand synthesis kit (Invitrogen, Cat: 1451351). qRT-PCR was performed on an AB 7500 (Applied Biosystems) real-time PCR machine and cDNA was amplified in duplicates with Quantitech SYBR Green kit (Qiagen, Cat: 204143). Pre-designed primer sets were obtained from Qiagen for Homo sapiens NPHS1 (nephrin), KIRREL (neph1), NPHS2 (podocin), WT-1, CD2AP, SYNPO (synaptopodin) and GAPDH. Changes in the expression levels of the first six of these genes were normalized to the reference gene (GAPDH) using the ΔΔCT method^30^. The final data is presented as relative ratio of expression as compared to uncoated culture dishes.

### Microarray Experimental Design and Gene List Retrieval

We characterized the effects of substrate stiffness on the differential gene expression signatures of podocytes using microarray-based network analysis as previously described^31^. The microarray experiment was performed on an Illumina beadarray platform with Human HT-12 v4 (BD-103-0204). Podocytes were cultured on tissue culture dishes for 48 hours at all conditions (0.6 kPa, 2 kPa, 5 kPa, 13 kPa, control) and total RNA was extracted using RNeasy kit per manufacturer’s instructions. The raw fluorescence data with background noise removed were analyzed with Matlab using the bioinformatics toolbox. For this analysis, intensity values were quantile normalized and pairwise compared with the control, and the false discovery rate (FDR) was adjusted by the Benjamini and Hochberg method^32^. We selected the top 250 genes with consistent changes among triplicate samples relative to control. Using the differentially expressed gene lists from the 2 kPa and 5 kPa groups, a “podocyte mechanotransduction network” was constructed with the X2 K suite and the complete human protein-protein interactome with intermediate genes among the seed nodes^33^. Further enrichment analyses were carried out using the EnrichR suite^34^ with ChEA, Reactome, and Wikipathways databases (r.2016).

### Statistical Analyses

All experiments were repeated at least three times; for normally distributed data, means were obtained from three or more independent measurements. The data were presented as mean ± standard error of the mean. T-statistic, the ratio of the departure of a parameter from a hypothesized value over its standard error, was used in hypothesis testing to indicate statistical significance of normalized parameters. Statistical differences between two groups of cells were determined using unpaired two-sample t-test or one-way ANOVA followed by a post-hoc Tukey test, where appropriate. In all figures, a single asterisk indicates statistical significance with p < 0.05 and a double asterisk indicates p < 0.01.

## Results

### Podocyte mechanotransduction modulates cytoskeletal assembly, migration, and proliferation

**Figure 2:**
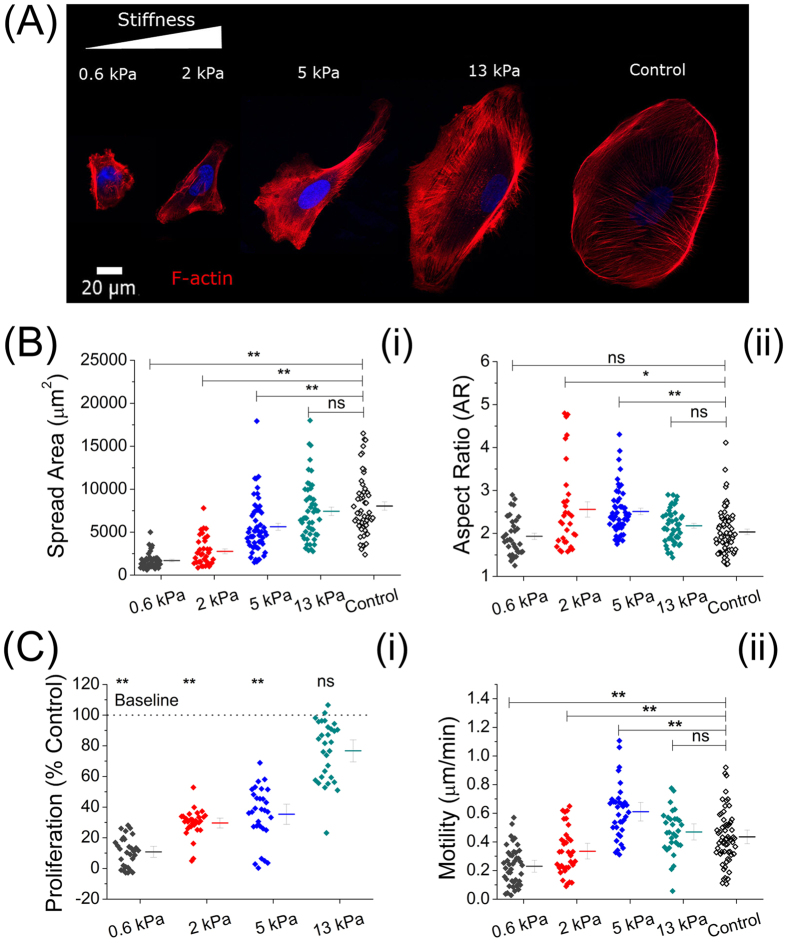
Podocyte cytoskeletal assembly, spreading, and cell proliferation are modulated by substrate stiffness. (**A**) Representative confocal microscope images of phalloidin-stained podocytes highlighting the actin cytoskeleton. Podocytes on the softest (0.6 kPa) gel spread poorly and were round. With increasing gel stiffness, podocytes were found to be more elongated with larger spread areas on the 2 kPa and 5 kPa gels. On the 13 kPa gel, podocyte geometry was isotropic, yielding similar contours to those cultured on tissue culture dishes (control). (**B**) Podocyte morphological characteristics (n > 30, each). (i) Podocyte spread area increased with increasing gel stiffness: 1692 ± 137 μm^2^ (0.6 kPa), 2766 ± 283 μm^2^ (2 kPa), 5635 ± 408 μm^2^ (5 kPa), 7528 ± 490 μm^2^ (13 kPa), the last of which was similar to control (8030 ± 351 μm^2^). (ii) Aspect ratio (AR) of podocytes on the softest (0.6 kPa) gel (AR = 1.9 ± 0.1) were significantly smaller than those on the stiffer gels (2 kPa: AR = 2.6 ± 0.2; 5 kPa: AR = 2.5 ± 0.1). However, the AR of cells on gels with the highest stiffness (13 kPa: AR = 2.2 ± 0.1) was similar to the softest gel (0.6 kPa) and to that of the control (AR = 2.0 ± 0.1). (**C**) Podocyte proliferation and motility (n > 30, each). (**i**) MTT assay showing podocyte proliferation as a percentage of control, which increases with increasing gel stiffness: 11 ± 3% (0.6 kPa), 30 ± 4% (2 kPa), 37 ± 5% (5 kPa), and 78 ± 6% (13 kPa). Results on the 0.6 kPa, 2 kPa, and5 kPa gels were significantly different from control (p < 0.01). (ii) Motility assay quantifying rate of migration of podocytes on gels with varying stiffness. Mean motility rate was observed to increase with increasing stiffness on soft to intermediate gels: 0.24 ± 0.04 μm/min (0.6 kPa), 0.35 ± 0.06 μm/min (2 kPa), and 0.61 ± 0.08 μm/min (5 kPa). On the 13 kPa gel, motility rate was 0.48 ± 0.06 μm/min, which was similar to that of control, 0.46 ± 0.05 μm/min.

We first tested whether gel stiffness can regulate the morphology, spreading, migration, and proliferation of podocytes. Figure 2a shows representative morphologies of podocytes after 5 days of culture. Conditionally immortalized human podocytes^29^ were cultured on gel-covered coverslips and bare coverslips as control. While gelatin-mTG gels can be used without additional ECM coating, all coverslips were coated with collagen I (5 μg/cm^2^) for this portion of the study. Changes in cellular morphological features were observed by performing stack imaging on phalloidin to demonstrate podocytes’ cytoskeletal network (2-D slices and vertical profiles are shown in Figure S2). The spread area was smallest for podocytes on the softest gel (0.6 kPa) and increased continuously with increasing gel stiffness (Fig. 2b-i). The inability to spread on soft gels (<1 kPa) is consistent with observations for other cell lines^35^,^36^. Podocyte shape and, in particular, front to back elongation, was also found to be modulated by substrate stiffness (Fig. 2b-ii). In contrast to spread area, however, while podocytes on both the softest and stiffest substrates (i.e. 0.6 kPa and 13 kPa gels) had average aspect ratios (AR) close to 2, those on substrates of intermediate stiffness (2–5 kPa) were more elongated (Avg. AR ≥ 2.5). This non-monotonicity is consistent with a similar finding for cardiomyocytes as a function of substrate stiffness^37^. On the stiffest gel (13 kPa), podocytes underwent isotropic spreading, and the cytoskeletal organization became similar to that of podocytes cultured on collagen coated culture dishes (control). The gradual appearance of stress fibers as gel stiffness increased from 0.6 kPa to control indicated cytoskeletal remodeling induced by gel stiffness^38^,^39^.

Differentiated podocytes are characterized by a lower proliferation rate compared with de-differentiated ones^40^. Figure 2c-i shows the results of the MTT assay that measures proliferation rate. Podocytes on 0.6 kPa, 2 kPa and 5 kPa gels showed substantially lower proliferation (11%, 30%, and 37% of control, respectively), while the 13 kPa gel showed relatively high cell proliferation (78% of control). As shown in Fig. 2b-ii, this softness-induced growth inhibition coincided with modulation of motility. Motility was significantly reduced compared to the control on the 0.6 kPa (0.24 ± 0.04 μm/min) and the 2 kPa (0.35 ± 0.06 μm/min) gels and enhanced on the 5 kPa gels (0.61 ± 0.08 μm/min). The motilities were found not to be significantly different between the 13 kPa gel and control (0.48 ± 0.06 μm/min and 0.46 ± 0.05_ _μm/min, respectively). *In vitro* podocyte motility is commonly assessed as a surrogate for physiological function; it is thought that podocytes *in vivo* need to maintain a certain level of motile capacity, balancing a high degree of spreading with modulatable migration^41^,^42^. Results showing statistically significantly altered migratory rates on soft gels relative to control confirm that gel stiffness has a regulatory effect on podocyte migration.

### Molecular quantification indicates differentiation phenotype on gels with near physiological stiffness

We next investigated the transcriptomic and proteomic effects of substrate stiffness and whether substrate stiffness similar to that of *in vivo* glomerular tissues would induce biochemical specialization. To do so, we measured expression of six key mRNA markers by RT-PCR. We selected these genes based on prior literature, as they are accepted to be associated with podocyte specificity, differentiation, and/or function^43^,^44^,^45^,^46^, and with a special focus on components of the mature slit diaphragm (Neph1, CD2AP, podocin, and nephrin), as discussed in a recent review^47^.

**Figure 3:**
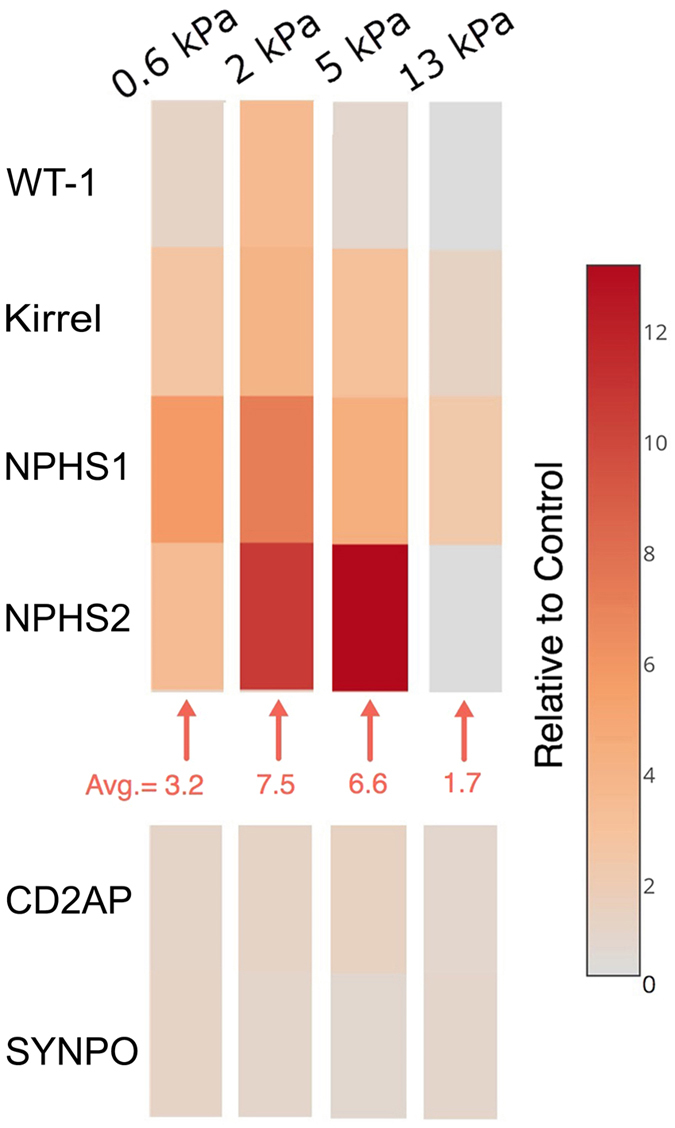
Gene expression indicates podocyte mechanotransduction can induce a pro-differentiation phenotype. Expression levels of six genes associated with podocyte differentiation and/or function were measured using qRT-PCR to test podocyte phenotype, namely: WT-1, KIRREL, SYNPO, NPHS1, NPHS2 and CD2AP. Colors in the heatmap represent the ratios of differential expression versus control. Four of the six genes showed upregulation on gels with different elastic moduli. Average fold-changes for these genes were 3.2, 7.5, 6.6, and 1.7 on the gels of 0.6 kPa, 2 kPa, 5 kPa, and 13 kPa, respectively.

Quantitative bioassays of immortalized human podocytes were performed after 48 hours of culture on gels of varying stiffness versus a standard culture dish (control), all coated with type I collagen. The expression levels of these six markers, relative to control, on all four gels are shown in Fig. 3. Compared to control cells, podocytes cultured on gelatin-mTG substrates increased expression of WT-1 (WT-1), Kirrel (neph1), NPHS1 (nephrin) and NPHS2 (podocin). The highest upregulation was found on the 2 kPa and 5 kPa gels, while the 13 kPa gel showed minimal increase in mRNA expression of these markers. CD2AP (CD2AP) and SYNPO (synaptopodin) did not show significant changes with varied substrate stiffness.

**Figure 4:**
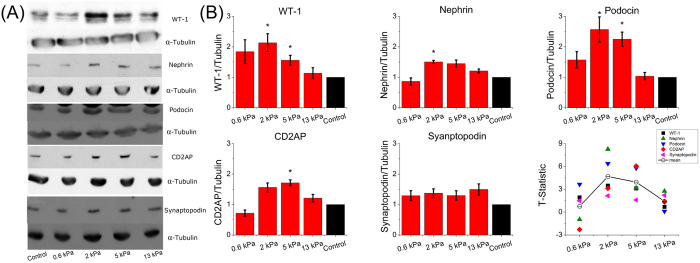
Stiffness-mediated differentiation phenotype induces upregulation of podocyte-specific functional proteins. (**A**) Representative images of Western blots for five proteins critical for physiological function of podocytes. Images of the complete blots are shown in Figure S3. (**B**) Quantification of kidney podocyte protein markers. Highest upregulation of WT-1 was found on the 2 kPa gel (2.2 ± 0.3). Nephrin, podocin, and CD2AP showed similar trends. Synaptopodin did not show this trend and there was no statistical difference between softer gels and control. T-statistics showed statistically significant changes of the ratios of five protein markers after normalization by control (two-tails, α = 0.05). The average t-statistics were 4.5 for the 2 kPa gel and 4.0 for the 5 kPa gel, which were significantly different relative to the control. Those for the 0.6 kPa and 13 kPa gels were 0.8 and 1.5, and the differences were not significant.

We next examined the protein expression levels for five of these markers for podocytes cultured on the gelatin-mTG gels, quantified as normalized ratios to control using Western blots (Fig. 4a, Figure S3). WT-1, a protein critical to podocyte maturation, as well as the three tested proteins associated with mature slit diaphragms (nephrin, podocin and CD2AP) were found at higher levels for cells cultured on the 2 kPa and 5 kPa gels. Podocytes on the 2 kPa gel had an average increase of 2.0 ± 0.2 times the concentration of these four proteins relative to the control group (p < 0.05), while podocytes on the 5 kPa gel had 1.9 ± 0.2 times the averaged concentrations. The 0.6 kPa and 13 kPa gels did not show significant increases in concentration. Staining for two of these proteins (podocin and nephrin) was done, and no change in distribution of the proteins was apparent as a function of underlying substrate stiffness (Figure S4). As at the gene expression level, at the protein expression level synaptopodin was present for podocytes cultured on all substrates and was not found to vary significantly with substrate stiffness. Representative Western blot images are shown in Fig. 4a and uncropped versions of these blots are shown in Figure S3.

We examined the t-statistics, as shown in Fig. 4b, to determine the persistent deviation of each gel from the control as determined by all Western blot assays combined, with the assumption of normality. The null hypothesis was that the Western blot ratios of any marker would average to 1. Average t-statistics for the 0.6 kPa and 13 kPa gels were 0.8 and 1.5, respectively, while the average t-statistics scores for the 2 kPa and 5 kPa gels were 4.5 and 4.0, respectively. Both scores indicate significant difference from the control (N > 3, two tails). Thus, measurement of protein expression confirms that the 2 kPa and 5 kPa gels are most effective in upregulation of proteins that are key in podocyte differentiation and function. This optimal range is in agreement with the *in vivo* stiffness of the glomerulus.

### Extent of mechanotransduction is extracellular matrix independent

While gelatin-mTG substrates can be used for podocyte culture without ECM coating, gelatin is not a component of native glomerular basement membrane, and it lacks the relevant secondary structures. As such, all above experiments were performed on type I collagen coated surfaces. We next examined whether the concentration or identity of the extracellular matrix protein coating of the gels contributed to the mechanotransduction effects by measuring protein expression on gels with different coatings. First, collagen I coating was repeated, but with density reduced from 5 μg/cm^2^ to 1 μg/cm^2^. Next, we investigated the effects of the three other ECM components of the GBM, namely type IV collagen (1 μg/cm^2^), laminin (1 μg/cm^2^) and fibronectin (1 μg/cm^2^), on the substrate stiffness response of podocytes. When coated on glass coverslips, the specific coating protein had no distinguishable effect on spreading area or cellular elongation (Figure S5).

**Figure 5:**
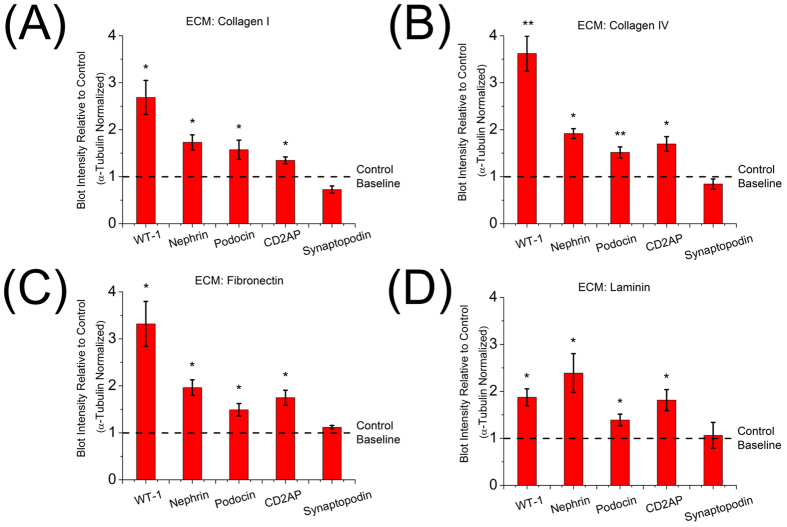
Extent of mechanotransduction is extracellular matrix independent ECM coating with **(A)** collagen I of reduced density from 5 μg/cm^2^ to 1 μg/cm^2^: Upregulation for five protein markers are 2.7 ± 0.3, 1.8 ± 0.1, 1.7 ± 0.2, 1.3 ± 0.1 and 0.7 ± 0.1 respectively, **(B)** collagen IV (1 μg/cm^2^): Relative expression levels of WT-1, nephrin, podocin, CD2AP and synaptopodin by Western blots were 3.7 ± 0.4, 1.9 ± 0.1, 1.5 ± 0.1, 1.7 ± 0.1, 0.8 ± 0.1 respectively, **(C)** fibronectin (1 μg/cm^2^): Relative expression levels of WT-1, nephrin, podocin, CD2AP and synaptopodin were 3.3 ± 0.4, 2.0 ± 0.1, 1.5 ± 0.1, 1.7 ± 0.1, 1.1 ± 0.1 respectively, **(D)** laminin (1 μg/cm^2^): Relative expression levels of WT-1, nephrin, podocin, CD2AP and synaptopodin were 1.8 ± 0.2, 2.4 ± 0.4, 1.4 ± 0.1, 1.8 ± 0.2 and 1.1 ± 0.2 respectively. In all cases, statistical significance indicated changes of the ratios of five protein markers after normalization by those of control (two-tailed t-test, α = 0.05).

For the study of podocyte gene expression as a function of ECM coating, the 2 kPa gel, which showed the highest t-statistics above, was used. As shown in Fig. 5a, reducing the density of the collagen I coating did not change the phenotype, with similar upregulation for WT-1, nephrin, podocin, and CD2AP. On average, their concentrations were 1.8 ± 0.2 times those of podocytes cultured on a culture dish, while synaptopodin was not found significantly different. On gels coated with collagen IV (Fig. 5b), we found that podocytes had 2.1 ± 0.2 times on average the concentrations of WT-1, nephrin, podocin, and CD2AP relative to control. Similarly, fibronectin (Fig. 5c) coating resulted in 2.1 ± 0.3 times the averaged concentrations. Laminin coating (Fig. 5d) yielded a similar ratio of 1.8 ± 0.2. Synaptopodin expression was not found to be significantly different from control for any coating. In sum, our findings show that the change of phenotype seen as a function of substrate rigidity was not due to a specific ECM coating density or component.

### Signaling pathways that are associated with podocyte differentiation are highly enriched on gels that approximate physiological stiffness

**Figure 6:**
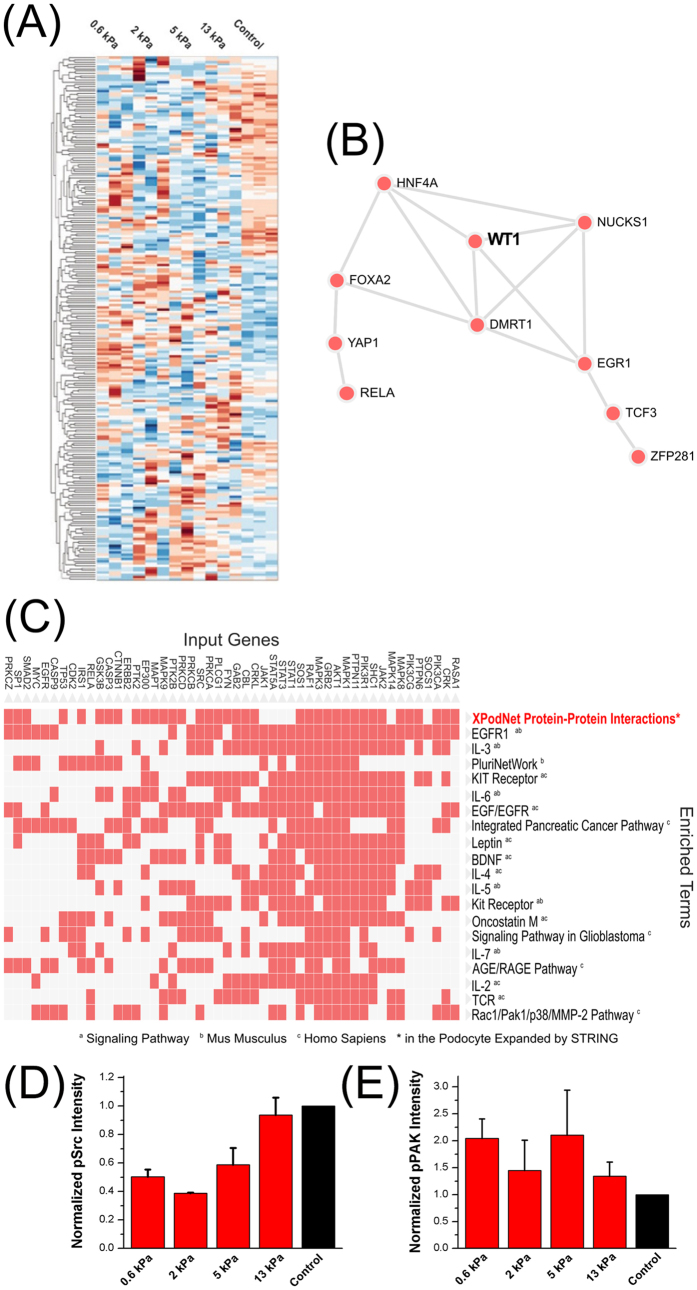
Pathways and networks contributing to stiffness-mediated podocyte differentiation. (**A**) Heatmap of the top 250 differentially expressed genes for podocytes cultured on gels of different moduli as measured by gene expression microarray. The complete list of genes and their respective fold changes are listed in Table S1. (**B**) Network representation of transcription factors that are enriched from the protein-protein interaction network shown in Figure S7 using the ChEA database. WT-1 was ranked among the top regulators of podocyte mechanosensing as a highly interconnected upstream transcription factor. Full names from the ChEA database are given in Table S2. (**C**) The clustergram demarcates the cluster of genes in the podocyte mechanotransduction network that were most commonly linked to the enriched terms within Wikipathways, which are sorted according to their z-scores. Enrichment analysis ranked the “PodNet: protein-protein interactions in the podocyte” as the top associated process for the podocyte mechanotransduction network. (**D**) Activity of Src, which was the central node of the podocyte mechanotransduction network, was highly dependent on substrate elasticity. The active form of Src in human podocytes was measured by Western blotting against phospho-Y416-Src that was normalized against α-tubulin (Normalized pSrc Intensity). (**E**) Activity of p21-activated kinase (PAK), which is a downstream effector of Src within the Rac signaling pathway, also depended on substrate elasticity. The active form of PAK in human podocytes was measured by Western blotting against phospho-T423-PAK1/2/3 that was normalized against GAPDH (Normalized pPAK Intensity).

We next measured gene expression using bead microarrays to identify mechanisms at the genetic level that may contribute to the observed phenotypic changes on the 2 kPa and 5 kPa gels. Using the top 250 differentially expressed genes from the 2 kPa and 5 kPa gels (Fig. 6a, Table S1), we constructed a podocyte mechanotransduction network using one intermediate within the complete human interactome. The resultant network had 381 nodes and 2,362 edges (Figure S6). The central node with the highest connectivity for the podocyte mechanoresponse was Src, which is known to play a critical role in integrin signaling and podocyte cytoskeletal integrity^48^. To validate this result, Western blotting for active Src (phospho-Y416) was performed for podocytes cultured on substrates of all stiffnesses and control, all coated with collagen I (Fig. 6d). This revealed that Src activity was indeed strongly modulated by substrate stiffness, as predicted by the network analysis.

Other highly connected nodes were EGFR, Shp2, SMAD2/3 and Erk1/2^49^,^50^,^51^,^52^, which have all been associated with aspects of podocyte biology. Besides Src, our enrichment analysis for the upstream kinases of interest (Figure S7a), such as Fyn and Lck, reflected increased association of podocyte-specific protein-protein interactions with mechanotransduction^53^,^54^. Moreover, several signaling pathways and upstream transcriptional regulators directly related to podocyte differentiation and physiological functions were found enriched within this podocyte network. One clear podocyte-associated signature, for example, was the upstream enrichment and interconnectedness for the transcription factor WT-1 (Fig. 6b), which is a master regulator for podocyte differentiation^54^,^55^. WT-1 was also found to be highly upregulated both at the transcript and proteomic levels on 2 kPa and 5 kPa gels. Most strikingly, Fig. 6c and Figure S7c show that the highest enriched term for the genes within this podocyte mechanotransduction network was “PodNet: protein-protein interactions in the podocyte” in Wikipathways (r.2016). 109 of the 381 nodes within our network were overlapping with the PodNet components, which was highly significant (adjusted p-value, 2.1 × 10^18^). The top cluster within this overlap along with the other enriched processes is also shown in the clustergram in Fig. 6c. These include EGFR1 and Rac1/Pak1/p38/MMP signaling pathways, all of which mediate podocyte physiology^50^,^56^, which further corroborates our findings that gel stiffness can regulate podocyte properties. We tested one of these predicted pathways, namely the Rac/PAK signaling pathway, by blotting for the active form of p21-activated kinase (PAK) family kinases phosphorylated at the threonine-423 residue^57^. Accordingly, we confirmed that PAK activity was also modulated by substrate stiffness (Fig. 6e), corroborating that the Rac pathway, which is downstream of Src, is involved in podocyte mechanotransduction.

The enrichment of some of the signaling pathways may account for the upregulation of podocyte-specific markers besides WT-1. For example, the NCI-Nature (r.2016) database (Figure S7b) showed that the highest enriched terms included Shp2, which associates with and enhances nephrin phosphorylation and is necessary for spreading of foot processes^49^. Additionally, VEGFR1/2 mediated signaling was enriched: it has been shown that differentiated podocytes express higher VEGFR2 mRNA levels than undifferentiated podocytes and the stimulation of this signaling pathway was proved to promote podocin upregulation, and nephrin/podocin/CD2AP interaction^58^,^59^. Based on these bioinformatics results, we conclude that mechanotransduction through sensation of substrate stiffness affects a wide variety of signaling pathways that are intimately related to podocyte differentiation and function.

## Discussion

As sclerosis or tissue softening often coexist with progressive symptoms in nephropathies^60^, it is important to understand whether kidney podoyctes respond to mechanical signals, and if so, to what extent their phenotype is modulated by changes in stiffness. In this study, we demonstrate use of a gelatin-based culturing platform providing a range of stiffness encompassing that of healthy glomerular tissue, with stiffness range explored here from 0.6 kPa to 13 kPa. Our results confirm that substrate stiffness can induce dramatic changes in podocyte phenotype. Podocytes cultured on different gels showed large morphological changes (Fig. 2a,b), with the greatest elongation on the 2 kPa and 5 kPa gels. There was also a significant difference in podocyte motility and proliferation on gels of 2 kPa and 5 kPa relative to stiffer substrates (Fig. 2c). Together this indicates that gel stiffness has regulatory effects on the phenotype of podocytes in the 2–5 kPa range, near the physiological stiffness of the glomerular basement membrane.

Molecular assays on gene products associated with podocyte specificity, differentiation, and/or function provided evidence that a pro-differentiation phenotype can be observed when substrate gel stiffness is between 2 kPa and 5 kPa. In particular, we found up-regulation of gene expression of WT-1 and three components of the mature slit diaphragm at stiffnesses similar to that of native healthy glomerulus. WT-1 is known to be a master upstream regulator of podocyte transcriptional networks that plays a critical role in podocyte maturation throughout renal development^55^,^61^. Furthermore, maintenance of WT-1 expression level is known to prevent physiological dysfunction^62^,^63^,^64^,^65^,^66^, further supporting a functionally improved *in vitro* pro-differentiation phenotype. Upregulation of WT-1 may suggest podocytes cultured on gels of intermediate stiffness are present in an environment closer to that of their *in vivo* counterparts than those cultured on typical culture substrates. Moreover, regulation of WT-1 as a function of substrate stiffness clearly shows that this critical protein is involved in a mechanotransduction network, and the evolution of glomerulus stiffness during development may play a role in the level of WT-1 at various points in podocyte maturation. Our enrichment analysis also independently highlighted WT-1 as an upstream transcriptional node (Fig. 6b), which provided additional evidence regarding its involvement in mechanotransduction. In contrast, synaptopodin, at both the gene and protein expression levels, was not found to vary significantly as a function of substrate stiffness. This indicates both that all culture conditions elicited podocyte specification and some degree of differentiation and that synaptopodin is not strongly coupled to mechanosensitive signaling pathways. Nephrin and podocin are essential proteins in the mature slit diaphragm and known to be involved in forming mechano-responsive complexes in human podocytes^67^. Their upregulated gene and protein expression implies high responsiveness to stiffness regulation. Gene expression of CD2AP, also involved in the mature slit diaphragm, was not greatly affected by stiffness but showed upregulation at the protein level. This suggests that mechanosensitive signaling pathways may play a role at the post-transcriptional regulation of this protein.

The Western blot assays for expression of various proteins described above and shown in Fig. 4 were repeated for substrates coated with different extracellular matrix proteins since the composition of the extracellular matrix can be a key contributor to cellular behaviors^68^. Differences persisted independent of coating type and density (Fig. 5). As collagen I, collagen IV, fibronectin and laminin all bind the integrin β_1_ receptor^69^, the mechanotransduction signaling pathway was present and activated in podocytes regardless of particular coating. No biochemical cue from specific ECM was found to be capable of altering or overwhelming the quantified effects of substrate stiffness. We note that even though the gelatin system described here is composed of denatured ECM protein, it presents varying levels of integrin binding sites even without additional surface coating and these endogenous sites may affect the basal levels of expression of proteins that are known to be regulated by integrin signaling.

Microarray expression profiling revealed that podocyte mechano-responsive genes are highly interconnected and converged on several signaling pathways centered on Src, a result that was validated through measurements of its activity levels through tyrosine phosphorylation of its kinase domain (Fig. 6d). In addition, other signaling pathways found can account for observed changes in the differentiated phenotype. Shown in the enrichment analysis outlined in Figure S7, these pathways include the leptin signaling pathway, which accounted for the reversibility of WT-1 in a mouse nephropathy model^70^; BDNF, which was shown to repair podocyte damage and restore the expression of nephrin^71^; and the EPO signaling pathway that has a renoprotective effect against loss of nephrin^72^. We tested the response of one of these pathways by assaying the activity of PAK, a downstream kinase in the Rac pathway that plays a key role in actin cytoskeletal remodeling in podocytes^73^, and showed that it is indeed mechano-responsive (Fig. 6e). Both Rac-activated PAK and Src family non-receptor tyrosine kinases have been shown to play independent critical roles in podocyte physiology^73^,^74^. Src has a complex and dynamic relationship with small GTPase Rac, playing roles in its activation, inactivation and spatial regulation^75^,^76^,^77^. While the exact mechanisms through which these pathways play a role in podocyte mechanotransduction is beyond the scope of this study, it is possible that Src-mediated activation of RacGAPs^78^ or their localization to the membrane^79^ may be responsible for the mechanoresponse of the Rac pathway in kidney podocytes.

The gelatin-mTG substrate system with tunable stiffness employed in this study served as an ideal tool to determine the relationship between GBM stiffness and podocyte biology. These findings may help clarify aspects of pathogenesis of kidney disease. For example, thickening of GBM is an early sign of diabetic nephropathy, prior to podocyte injury^80^,^81^. We suggest that changes of GBM stiffness due to such thickening could be a contributing factor for podocyte injury in diabetic nephropathy and that this model gelatin system may be a useful tool for investigating and testing drugs for kidney disease. Given that the podocytes cultured on gels with stiffness near that of healthy GBM had higher levels of gene and protein expression associated with the mature slit diaphragm, the signature of terminal podocyte differentiation, our study also provides guidance for building better *in vitro* culture systems for podocytes, which has been a long-standing challenge^40^.

## Conclusion

In this study, we used the natural enzyme microbial transglutaminase to crosslink gelatin and construct a stiffness adjustable cell culture platform. It was found that substrate stiffness regulated podocyte spreading, migration, and proliferation. Elevated expression of markers of podocyte specificity, differentiation, and function was found both at the transcriptome and proteome level for podocytes cultured on substrates of intermediate stiffness (2–5 kPa), which is close to those of *in vivo* renal glomerular tissues, whereas both softer and stiffer substrates of identical biochemical composition (e.g. the 0.6 kPa and 13 kPa gels) did not induce this phenotype. This differentiated phenotype is characterized by high upregulation of gene and protein expression critical for kidney podocyte physiological function. This phenotype was found to be independent of the particular ECM composition. Network analysis using the top differentially expressed genes from the intermediate stiffness substrates enriched for several key signaling pathways that are related to podocyte differentiation and physiological function, further implicating mechanotransduction as a key player in podocyte biology. The gelatin-mTG platform, with variable stiffness over a range consistent with that seen for many soft tissues *in vivo*, may have important impact not only on nephrology research but also for studies of, for example, liver cells and neurons.

## Additional Information

**How to cite this article**: Hu, M. *et al*. A biomimetic gelatin-based platform elicits a pro-differentiation effect on podocytes through mechanotransduction. *Sci. Rep.* **7**, 43934; doi: 10.1038/srep43934 (2017).

**Publisher's note:** Springer Nature remains neutral with regard to jurisdictional claims in published maps and institutional affiliations.

## Supplementary Material

Supplementary Information

## Figures and Tables

**Figure 1:**
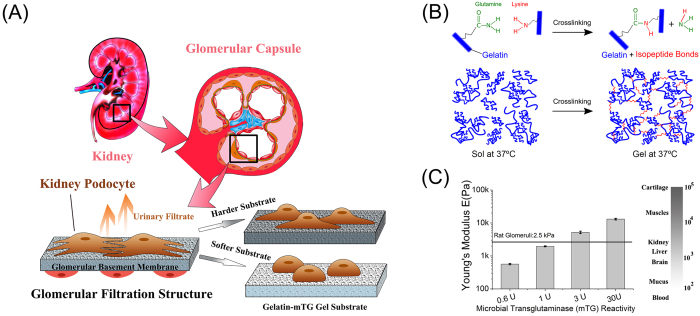
Stiffness-tunable gelatin-mTG hydrogel provides an ideal platform to study kidney podocyte mechanotransduction. (**A**) Schematic representation of human podocyte study using tunable stiffness gels to characterize podocyte phenotype. The cross-section of a kidney and its main functional unit, i.e. the glomerular capsule are shown (top). The tissue structure inside the glomerular capsule is framed to highlight the location of the glomerular filtration barrier, for which podocytes are essential. Podocytes (in brown) interdigitate with neighboring podocytes to form the slit diaphragm. The glomerular basement membrane provides mechanical support for podocytes. Capillary endothelial cells (in red) are separated from podocytes by the basement membrane. Mutations in glomerular basement membrane components and physical structure are found in many nephropathies, which are correlated with changes in glomerular tissue stiffness. By using a gel system with tunable stiffness, podocyte mechanotransduction can be analyzed. (**B**) Illustration of (top) the transglutamination reaction used for gelatin crosslinking, and (bottom) a schematic of the resulting crosslinked gelatin network. Microbial transglutaminase initiates the formation of covalent isopeptide bonds between lysine and glutamine residues on gelatin molecules. (**C**) Young’s moduli of gelatin-mTG gels crosslinked by four enzyme concentrations (0.6U, 1U, 3U and 30U) as measured by oscillatory rheology. At right, a scale demonstrating tissue stiffness of various human organs and soft tissues is shown.
