# Supplementary material for: A biomimetic gelatin-based platform elicits a pro-differentiation effect on podocytes through mechanotransduction
Source: Sci Rep. 2017 Mar 6;7:43934. doi: 10.1038/srep43934 (PMC5338254; doi:10.1038/srep43934)
Supplement: Supplementary Information [file srep43934-s1.docx]

**Supporting Information**

**A biomimetic gelatin-based platform elicits a pro-differentiation effect on podocytes through mechanotransduction**

Mufeng Hu^1,#^, Evren U. Azeloglu^2,#^, Amit Ron^1^, Khanh-Hoa Tran-Ba^3^, Rhodora C. Calizo^2^, Iman Tavassoly^2^, Smiti Bhattacharya^1^, Gomathi Jayaraman^2^, Yibang Chen^2^, Vera Rabinovich^2^, Ravi Iyengar^2,*^, James C. Hone^1,*^, John C. He^2,4,*^, Laura J. Kaufman^3,*^

^1^ Department of Mechanical Engineering

Columbia University

New York, NY 10027, USA

^2^ Department of Pharmacological Sciences

and Systems Biology Center New York

Icahn School of Medicine at Mount Sinai

New York, NY 10029, USA

^3^ Department of Chemistry

Columbia University

New York, NY 10027, USA

^4^ Division of Nephrology, Department of Medicine

Icahn School of Medicine at Mount Sinai

New York, NY 10029, USA

# Co-first authors

* Equal Contributions, joint senior authors

*Address correspondence to:*

Laura J. Kaufman, Ph.D.

Department of Chemistry

Columbia University

New York NY 10027

Phone: 212-854-9025

Fax: 212-932-1289

e-mail: [ljk15@columbia.edu](file:///C:\Users\laura\AppData\Local\Temp\ljk15@columbia.edu)

**or**

John Cijiang He, M.D., Ph.D.

Division of Nephrology, Department of Medicine

Icahn School of Medicine at Mount Sinai

New York, NY 10029

Phone: 212-659-1703

Fax: 212-241-0389

e-mail:[cijiang.he@mssm.edu](mailto:cijiang.he@mssm.edu)

In this supporting information, we provide additional figures and information on the rheology measurements, fluorescence imaging, and gene and enrichment analysis of podocytes on gelatin gels. Figure S1 shows representative rheology measurements used to determine moduli of the gels. Figure S2 depicts 3D stack profiles of fluorescently labelled podocytes on gels with varying stiffnesses. Figure S3 shows full Western blots associated with the cropped images and quantifications shown in Figs. 4 and 6 in the main text. Figure S4 shows immunofluorescent images of nephrin and podocin of podocytes on gels with varying stiffnesses. Figure S5 shows representative images of podocyte morphology on substrates with different ECM coating. Figure S6 shows the podocyte mechanosensing network identified through combined microarray and network analysis and Figure S7 provides the enrichment analyses for this network. Table S1 lists the differentially expressed genes shown in Fig. 6a. Table S2 provides additional details for the network representation of transcription factors shown in Fig. 6b.


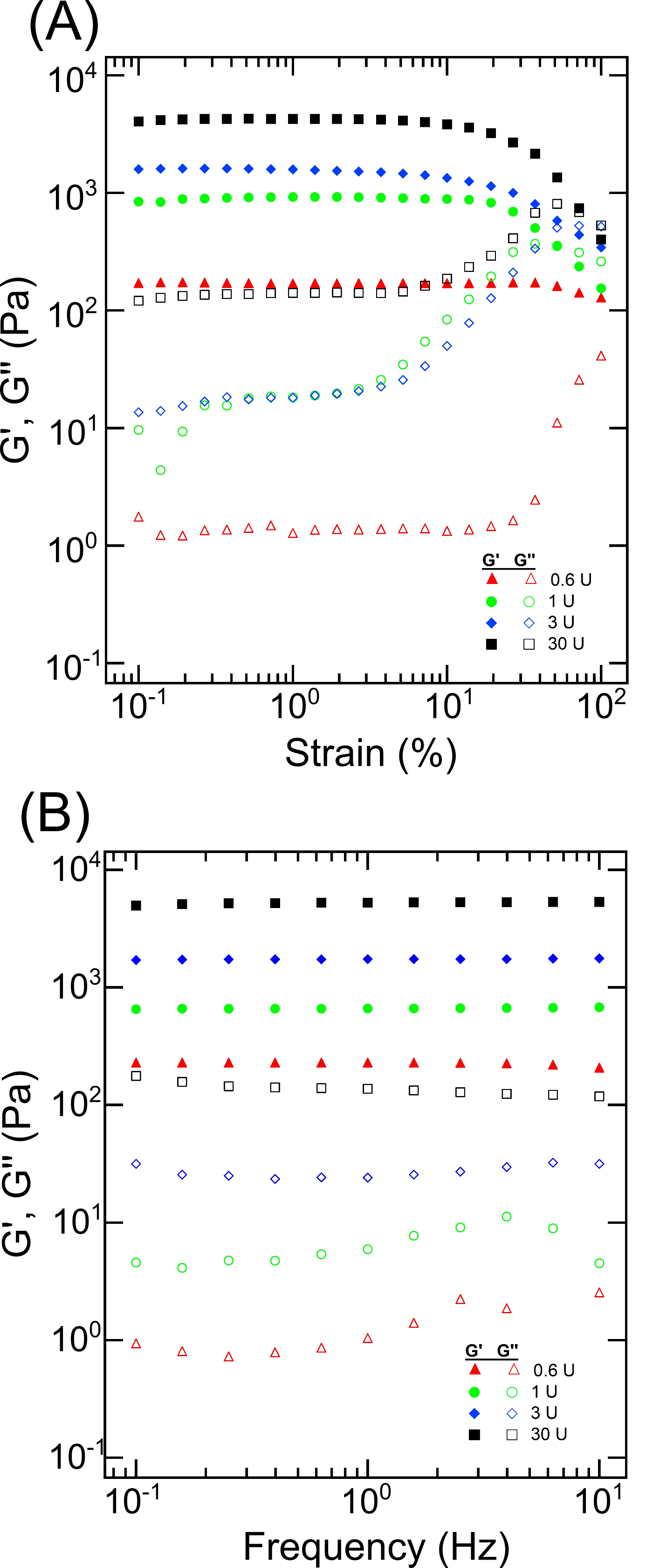


**Figure S1: Rheology Measurements**

(A) Strain sweep measurements performed with strain amplitude γ = 0.1% – 100% and fixed frequency f = 1 Hz to determine the linear regime of γ. (B) Representative frequency dependent measurements performed at γ = 1% from which shear storage moduli $G^{'}$ and shear loss moduli $G^{''}$ of gels with different enzyme reactivity (0.6U, 1U, 3U, 30U) were obtained.


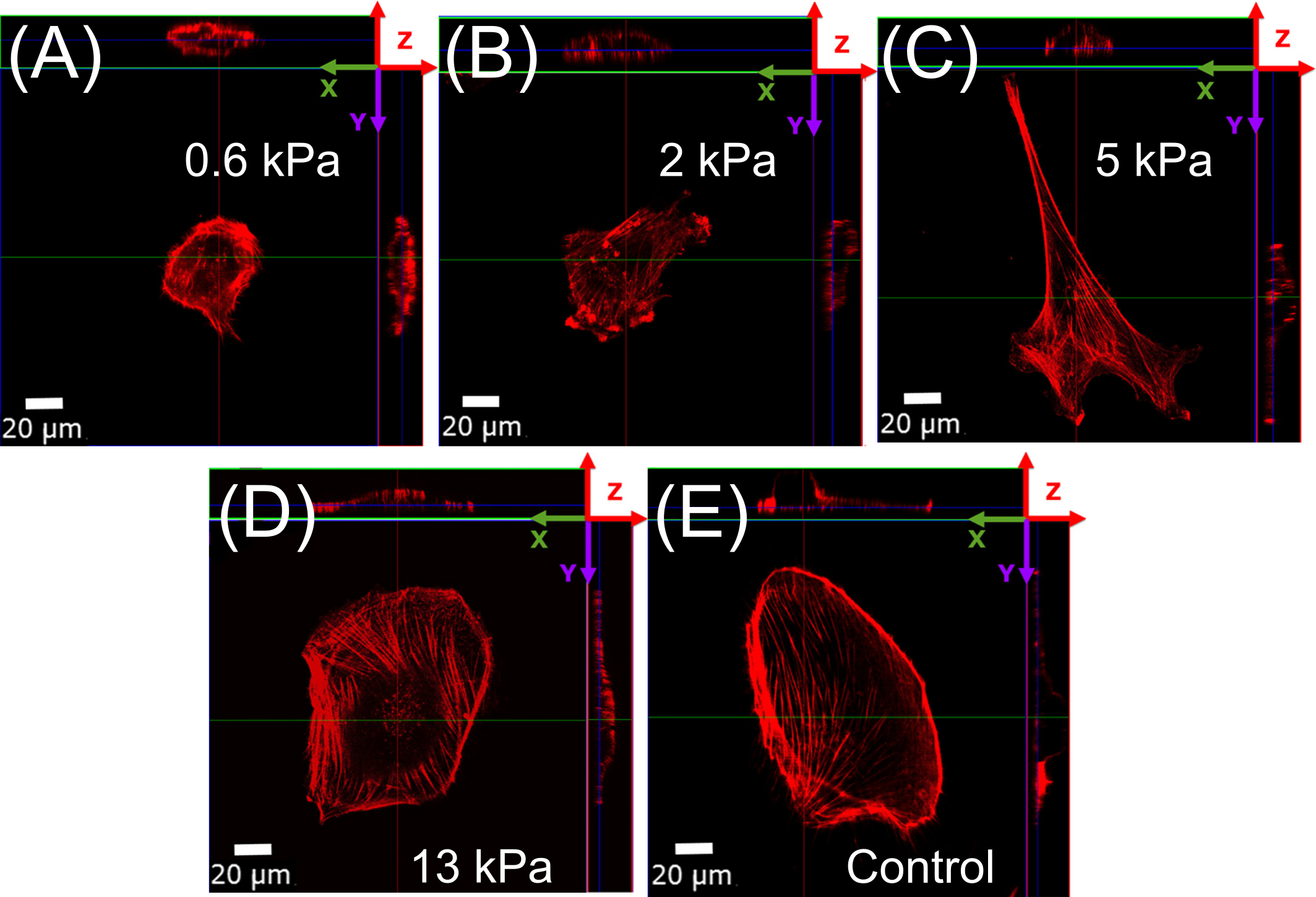


**Figure S2: 3D profiles of podocytes on different gels by stack imaging**

Representative images of podocytes cultured on 0.6 kPa, 2 kPa, 5 kPa, 13 kPa gels and control. Phalloidin staining revealed gradual changes of podocyte cytoskeletal organization with gel stiffness. The vertical cross sections of podocytes on the 0.6 kPa gel showed the podocytes created a slight indentation in this gel.

**Figure S3: Complete scans of Western blots**

Complete Western blots associated with the cropped images shown and quantified in Figs. 4 and 6 in the main text. We note that for podocin, quantification was performed for all bands in the 42kDa region rather than just the single band shown for display in Fig. 4 in the main text.


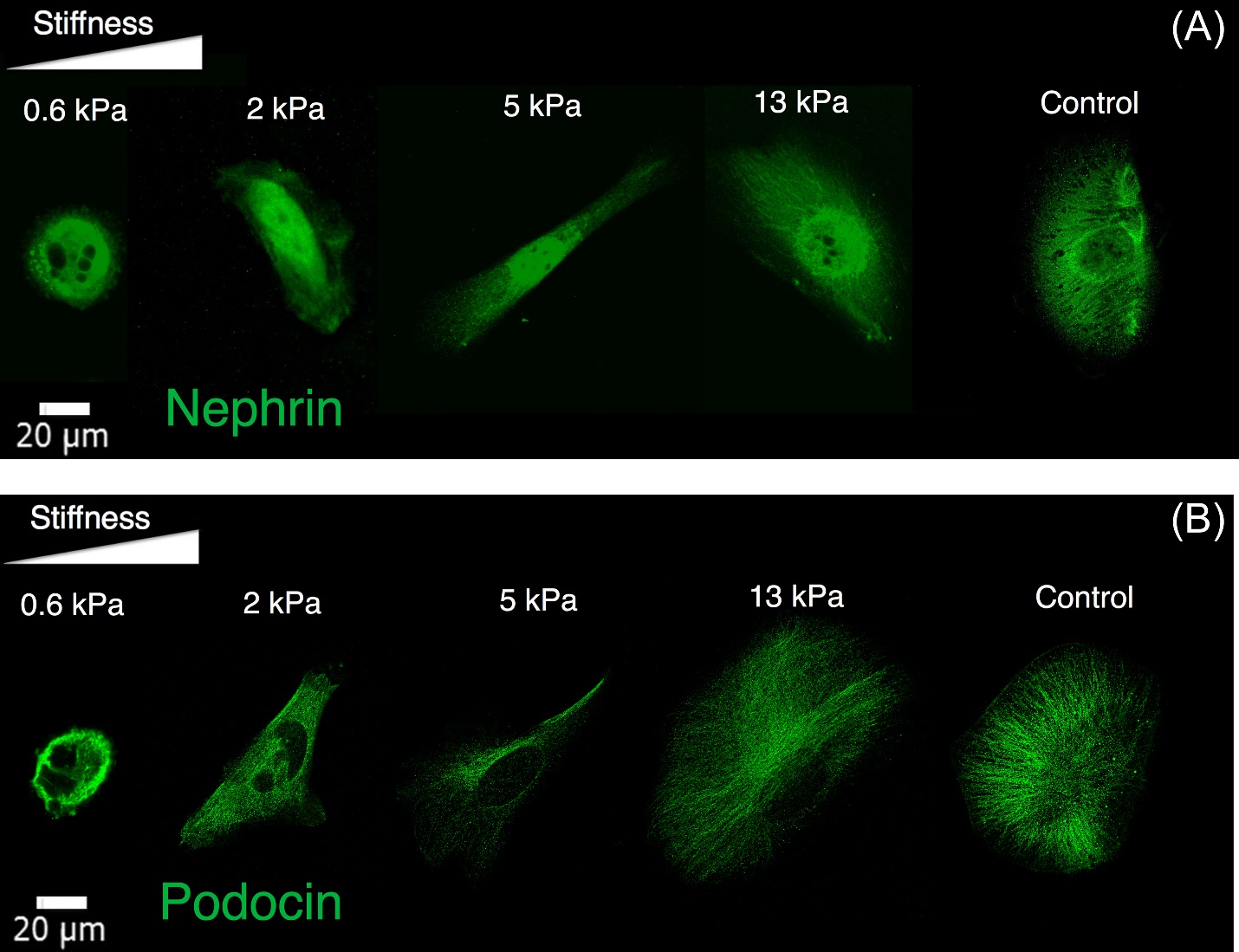


**Figure S4: Immunofluorescent images of nephrin and podocin in podocytes on different gels**

Representative immunofluorescent images of (a) nephrin and (b) podocin for podocytes cultured on 0.6 kPa, 2 kPa, 5 kPa, 13 kPa gels and control. No localization of the two proteins within the cells was found as podocyte morphology underwent gradual changes.


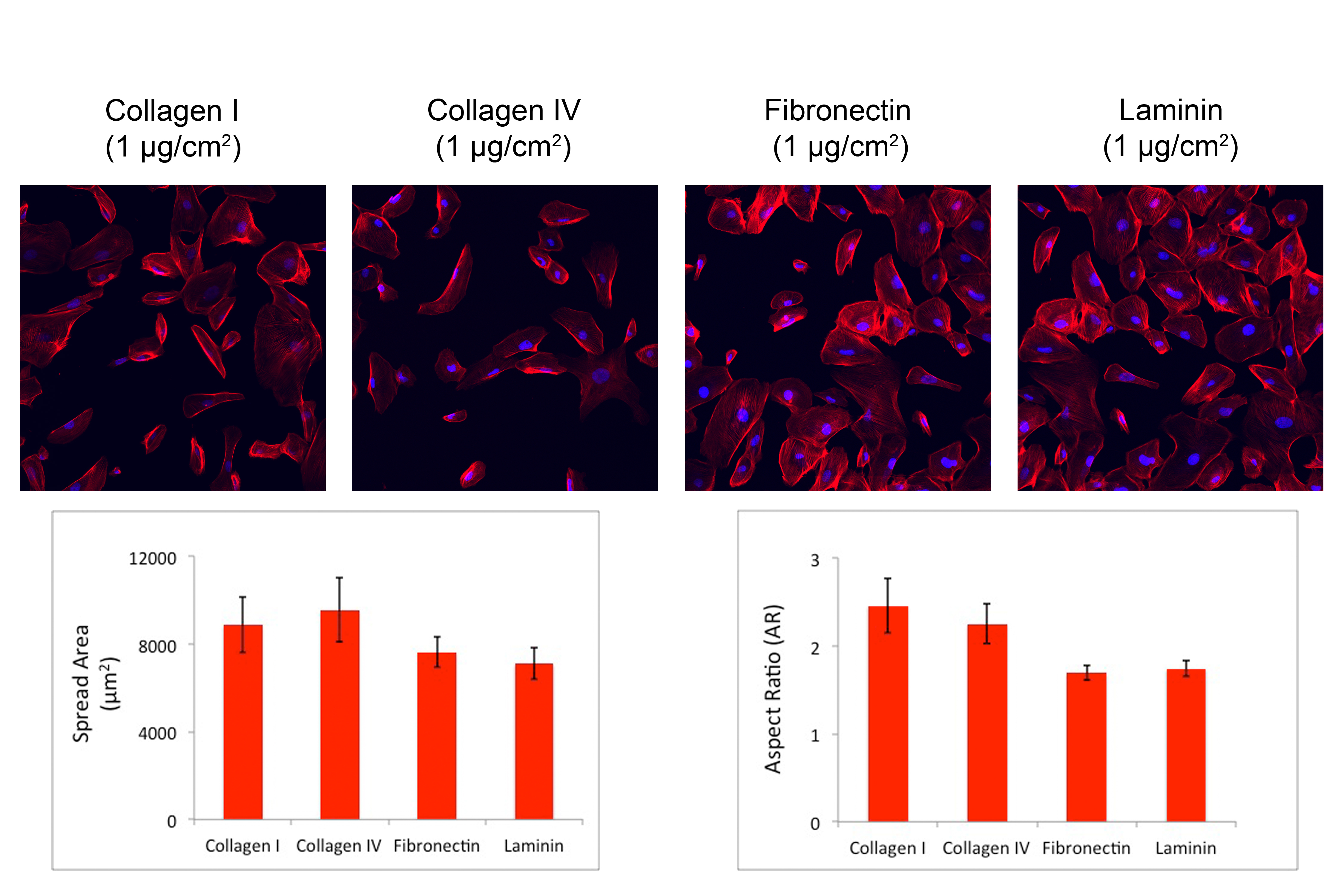


**Figure S5: Podocyte phenotype sensitivity to specific ECM coating protein**

(top) Representative images of podocytes cultured on glass coverslips coated with 1 μg/cm^2^ collagen I, collagen IV, fibronectin, and laminin show that cell morphology is not sensitive to particular coating protein. (bottom) Quantitative analysis shows similar spreading areas and aspect ratios for podocytes cultured on glass coverslips regardless of coating protein.


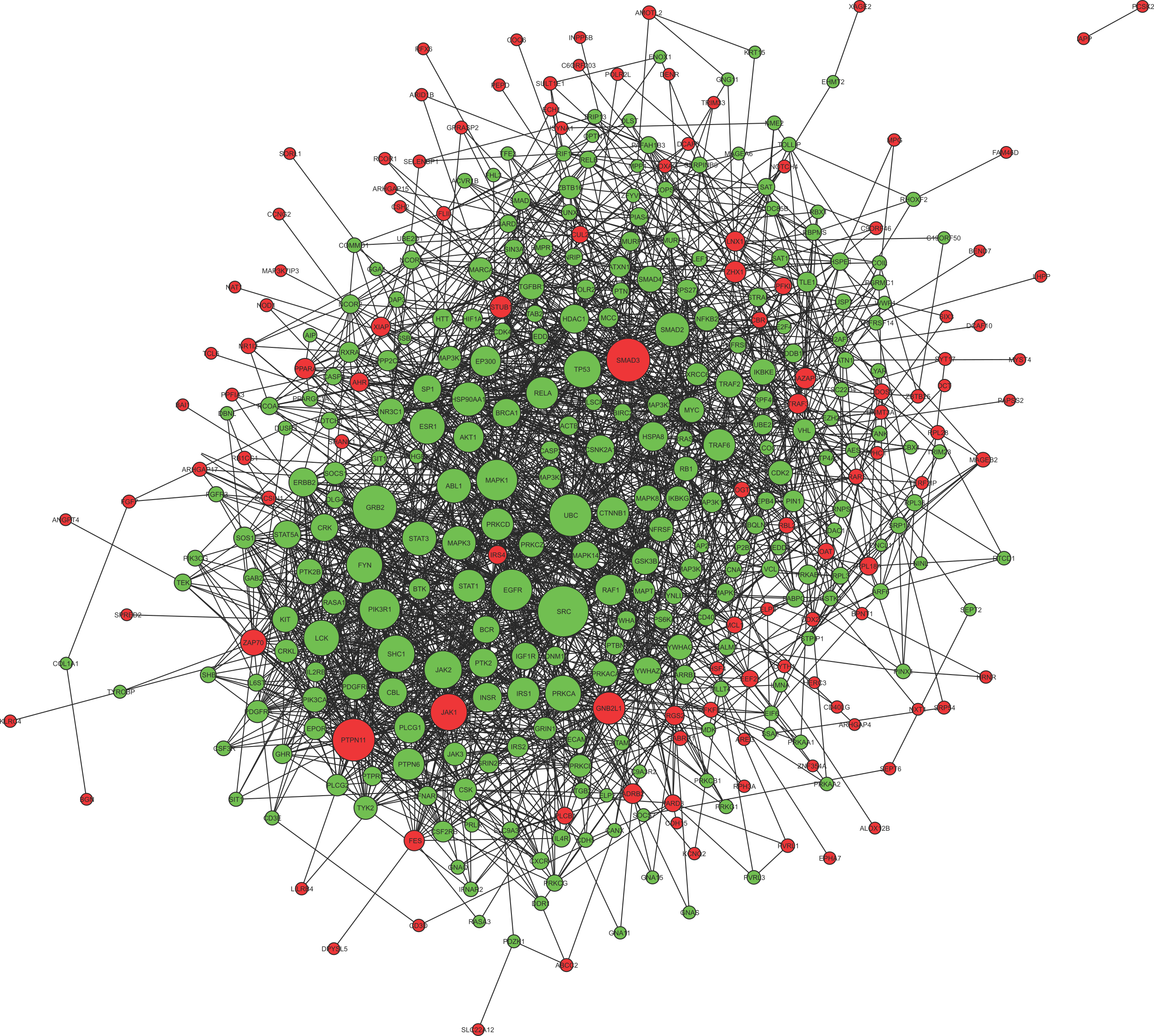
**Figure S6: Protein-protein interactome network of mechanoresponse**

As the milieu with highest podocyte differentiation, differentially expressed genes from the 2 kPa and 5 kPa gels (red nodes) versus control were used to construct a protein-protein interaction network by any intermediate (green nodes) between differentially expressed genes. The size of a node within this “podocyte mechanosensing network” represents the connectivity of that node.


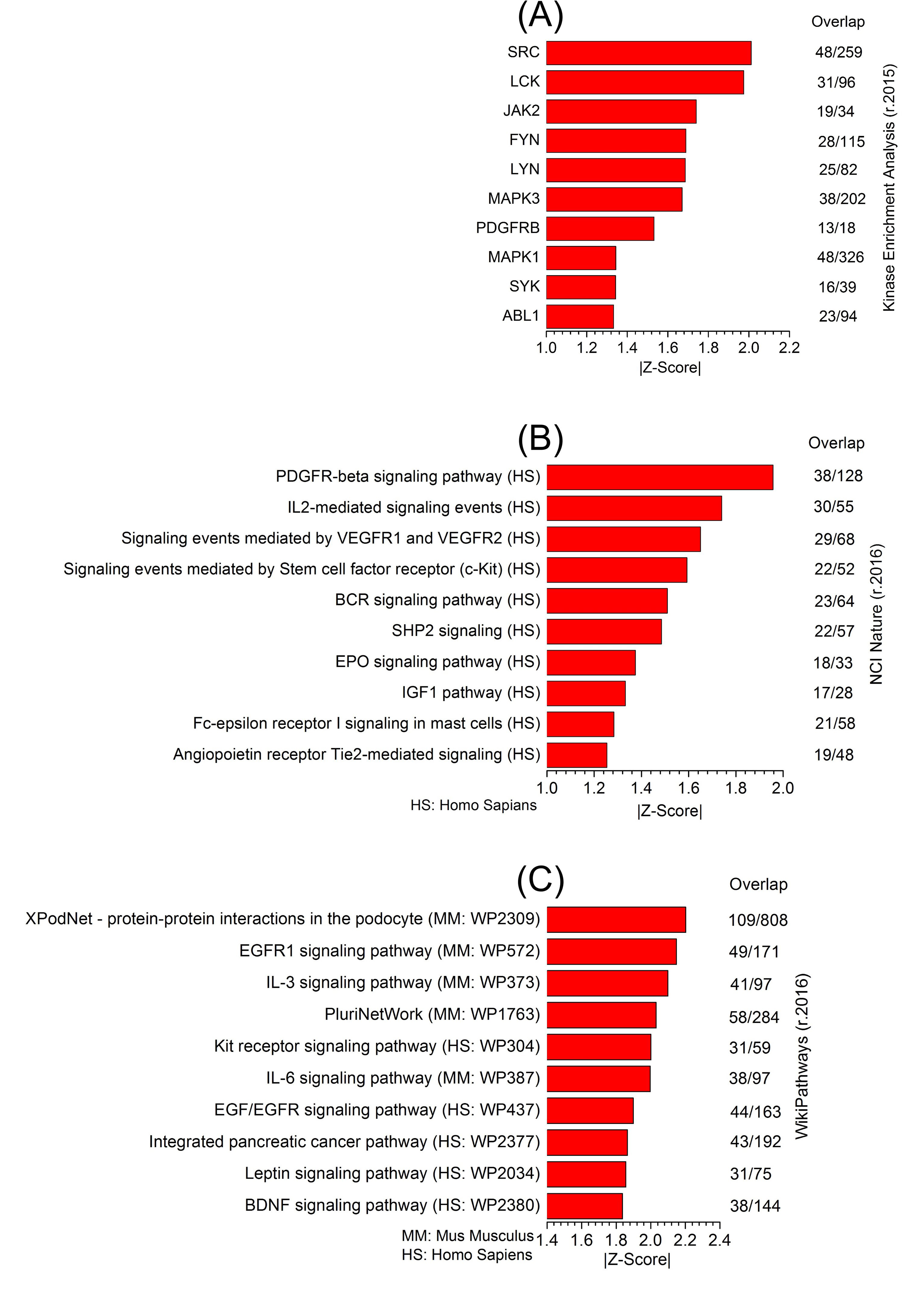
**Figure S7: Enrichment analyses using the podocyte mechanosensing network**

Top ranking enrichment terms according to **(A)** kinase enrichment analysis, **(B)** NCI Nature pathways, and **(C)** Wikipathways. The enriched term list for Wikipathways is a partial re-plot of the clustergram y-axis from Figure 6c, where the top 20 enriched terms were displayed without the total overlap counts. All analyses herein were carried out with the EnrichR suite using the components of the protein-protein interaction network that we termed the podocyte mechanosensing network (Fig. S6). The enriched components are all ranked according to the absolute values of z-scores. Next to each component, the number of genes in the podocyte mechanosensing network (containing 380 genes) that overlap with the total number of genes in the particular pathway or network identified are shown.

| Table S1. Differentially expressed genes and their fold changes under different substrate stiffnesses. | | | | |
| --- | --- | --- | --- | --- |
| Gene Name | **0.6 kPa** | **2.0 kPa** | **5.0 kPa** | **13 kPa** |
| ABCC2 | 0.9 | 0.8 | 0.7 | 0.7 |
| ABCG2 | 1.2 | 1.6 | 1.4 | 1.4 |
| ACVR1C | 1.3 | 1.4 | 1.3 | 1.2 |
| AMOTL2 | 1.0 | 0.8 | 1.2 | 1.2 |
| ANGPT4 | 0.7 | 0.7 | 0.5 | 0.8 |
| ANGPTL6 | 1.0 | 1.5 | 0.9 | 0.8 |
| APOA2 | 0.7 | 0.7 | 0.9 | 0.6 |
| AREG | 0.9 | 0.6 | 1.2 | 0.9 |
| ARHGAP17 | 1.1 | 0.9 | 1.0 | 1.1 |
| ARID1B | 0.7 | 0.8 | 0.9 | 1.0 |
| ATP2C1 | 0.9 | 1.0 | 1.1 | 1.1 |
| ATP4B | 1.3 | 1.7 | 1.3 | 1.3 |
| BAT2D1 | 1.2 | 0.8 | 0.8 | 1.0 |
| BEST2 | 0.5 | 0.7 | 0.6 | 0.9 |
| BTN2A3 | 0.9 | 0.8 | 0.9 | 0.9 |
| C10ORF72 | 1.0 | 0.9 | 0.7 | 0.8 |
| C11ORF44 | 0.9 | 1.0 | 0.9 | 0.7 |
| C17ORF78 | 0.6 | 0.8 | 0.6 | 1.1 |
| C1ORF68 | 1.4 | 1.3 | 1.0 | 0.9 |
| C1QTNF8 | 0.8 | 0.9 | 0.8 | 0.9 |
| C20ORF106 | 1.2 | 1.1 | 1.2 | 1.5 |
| C5ORF35 | 0.9 | 1.1 | 1.2 | 1.0 |
| CAPNS2 | 0.8 | 0.7 | 0.8 | 0.7 |
| CATSPER4 | 1.2 | 1.4 | 1.1 | 1.1 |
| CCDC80 | 0.6 | 1.5 | 2.1 | 1.3 |
| CCNJ | 1.2 | 1.3 | 1.5 | 1.5 |
| CCNYL1 | 0.8 | 1.2 | 1.3 | 1.1 |
| CD22 | 1.1 | 1.1 | 1.1 | 1.4 |
| CD3D | 1.0 | 0.9 | 0.6 | 0.7 |
| CD8B | 0.8 | 0.9 | 0.9 | 0.9 |
| CDX1 | 0.8 | 1.1 | 0.7 | 0.8 |
| CILP | 1.0 | 1.1 | 1.2 | 1.3 |
| CLRN1OS | 0.7 | 0.7 | 0.8 | 1.0 |
| COLEC10 | 1.1 | 0.9 | 0.6 | 0.9 |
| CPA6 | 1.2 | 1.4 | 1.3 | 1.3 |
| CPNE4 | 1.2 | 1.2 | 1.3 | 1.5 |
| CREBL2 | 1.0 | 1.0 | 1.0 | 1.1 |
| CUL2 | 0.9 | 0.8 | 0.9 | 1.0 |
| CXCL13 | 1.6 | 1.2 | 1.5 | 1.3 |
| CYP19A1 | 1.2 | 1.2 | 1.2 | 1.3 |
| DAPL1 | 1.0 | 0.6 | 1.0 | 0.7 |
| DAZAP2 | 0.9 | 0.9 | 0.9 | 1.0 |
| DCT | 1.6 | 1.6 | 2.0 | 1.8 |
| DDOST | 1.0 | 0.9 | 0.9 | 0.9 |
| DEFB119 | 0.9 | 0.8 | 1.0 | 0.9 |
| DENR | 0.9 | 0.9 | 0.9 | 1.0 |
| DHRS7C | 0.9 | 0.8 | 0.7 | 0.7 |
| DHTKD1 | 0.9 | 0.8 | 0.9 | 0.8 |
| DLEU7 | 0.8 | 1.0 | 0.9 | 0.9 |
| DNAH8 | 0.8 | 1.0 | 0.8 | 0.9 |
| DPYSL5 | 1.1 | 1.0 | 1.3 | 1.2 |
| DUSP23 | 1.0 | 1.2 | 1.4 | 1.1 |
| DUX5 | 0.7 | 0.9 | 0.8 | 1.1 |
| ECH1 | 1.2 | 1.0 | 0.7 | 0.8 |
| EFR3A | 1.1 | 0.8 | 0.8 | 0.9 |
| EPB41L3 | 0.9 | 1.0 | 1.3 | 1.1 |
| ERCC-00035 | 0.9 | 0.7 | 0.6 | 0.7 |
| ERCC-00134 | 1.6 | 1.2 | 1.3 | 1.3 |
| ERCC-00157 | 0.9 | 1.2 | 1.2 | 1.1 |
| ERVWE1 | 0.7 | 0.6 | 0.5 | 0.9 |
| F2 | 1.1 | 0.9 | 1.4 | 1.1 |
| FAM110C | 1.2 | 1.0 | 1.5 | 1.0 |
| FAM46D | 1.1 | 1.1 | 0.7 | 1.0 |
| FAM53C | 1.3 | 1.2 | 1.0 | 1.1 |
| FAM57B | 1.3 | 1.5 | 1.5 | 1.4 |
| FAM60A | 1.1 | 0.8 | 0.7 | 0.9 |
| FAM71F2 | 0.9 | 0.7 | 0.8 | 0.8 |
| FCGBP | 1.5 | 1.1 | 0.7 | 0.8 |
| FES | 0.9 | 1.3 | 1.2 | 1.1 |
| FGF20 | 1.3 | 1.4 | 1.2 | 1.2 |
| FGF3 | 0.5 | 1.0 | 0.9 | 0.7 |
| FLJ14816 | 0.6 | 0.7 | 0.7 | 0.6 |
| FLJ36070 | 1.1 | 1.0 | 1.0 | 1.1 |
| FLJ36701 | 0.8 | 0.8 | 0.9 | 0.6 |
| FMO1 | 1.0 | 1.1 | 1.0 | 1.3 |
| FRG2B | 0.9 | 1.2 | 1.2 | 1.2 |
| FSCN2 | 1.2 | 1.3 | 1.0 | 1.5 |
| GABRR1 | 1.2 | 1.3 | 1.0 | 1.1 |
| GAGE2B | 1.0 | 0.8 | 1.2 | 1.2 |
| GALNT13 | 1.3 | 1.2 | 1.3 | 1.7 |
| GIMAP8 | 0.9 | 0.9 | 1.0 | 1.0 |
| GJA8 | 0.8 | 0.6 | 0.9 | 0.8 |
| GLYATL2 | 0.6 | 0.5 | 0.8 | 0.7 |
| GNAT2 | 0.7 | 0.7 | 0.7 | 0.8 |
| GNB2L1 | 1.0 | 1.0 | 0.9 | 1.1 |
| GPR113 | 1.2 | 1.1 | 1.1 | 1.5 |
| GTF2IRD2P | 0.8 | 0.8 | 0.7 | 1.0 |
| H1FNT | 0.7 | 1.1 | 0.8 | 0.8 |
| H2BFWT | 0.8 | 0.8 | 0.9 | 1.0 |
| HCG27 | 1.2 | 1.1 | 1.6 | 0.8 |
| HP1BP3 | 0.9 | 0.8 | 0.8 | 1.0 |
| ICOSLG | 1.1 | 1.0 | 1.3 | 1.3 |
| IL16 | 1.2 | 1.3 | 1.4 | 1.6 |
| INSM2 | 0.7 | 1.0 | 0.8 | 0.7 |
| KCNIP4 | 1.2 | 1.2 | 1.2 | 1.2 |
| KCNK10 | 1.2 | 1.0 | 1.1 | 1.2 |
| KIAA0261 | 0.9 | 0.8 | 0.8 | 0.9 |
| KIAA1033 | 1.0 | 0.8 | 0.9 | 1.0 |
| KIF21B | 1.1 | 1.1 | 0.9 | 1.2 |
| KLRC4 | 1.1 | 1.3 | 1.0 | 1.1 |
| KRTAP10-9 | 0.7 | 0.8 | 0.7 | 0.8 |
| KRTAP20-3 | 0.6 | 0.6 | 0.9 | 0.8 |
| KRTAP5-11 | 1.0 | 1.0 | 1.2 | 1.1 |
| LCAP | 1.2 | 1.0 | 1.2 | 1.0 |
| LCT | 0.8 | 0.9 | 1.1 | 0.8 |
| LGI2 | 0.9 | 0.9 | 0.7 | 0.8 |
| LMAN2 | 0.8 | 1.2 | 1.3 | 1.1 |
| LOC162632 | 0.5 | 0.9 | 1.0 | 0.8 |
| LOC401296 | 1.5 | 1.2 | 1.1 | 1.4 |
| LOC644936 | 0.8 | 1.0 | 1.3 | 1.1 |
| LOC650293 | 0.6 | 0.5 | 0.5 | 0.5 |
| LTC4S | 1.2 | 0.9 | 1.0 | 0.7 |
| LYPD4 | 1.2 | 1.2 | 1.0 | 1.6 |
| MAGEB2 | 1.1 | 0.8 | 1.0 | 0.9 |
| MAP3K7IP3 | 1.0 | 0.8 | 0.7 | 0.8 |
| MBTPS2 | 0.8 | 1.0 | 0.8 | 0.9 |
| MCL1 | 0.9 | 1.2 | 1.0 | 1.0 |
| MEIG1 | 1.2 | 1.3 | 1.1 | 1.1 |
| MGAT2 | 0.8 | 1.1 | 1.3 | 1.1 |
| MIR1915 | 0.7 | 1.0 | 1.0 | 0.8 |
| MIR449C | 0.7 | 1.0 | 0.8 | 0.8 |
| MIR563 | 1.0 | 1.4 | 1.3 | 1.1 |
| MIR595 | 1.5 | 1.4 | 1.4 | 1.4 |
| MIR597 | 0.8 | 0.8 | 0.7 | 1.0 |
| MIR617 | 1.1 | 0.6 | 0.8 | 0.9 |
| MIR944 | 0.9 | 0.6 | 0.8 | 0.8 |
| MPPED1 | 1.3 | 1.1 | 1.2 | 1.4 |
| MRGPRX3 | 0.7 | 0.8 | 0.7 | 0.8 |
| MRPS18A | 1.2 | 1.0 | 0.9 | 1.1 |
| MYH6 | 1.3 | 0.8 | 1.2 | 1.3 |
| MYOM1 | 1.3 | 1.4 | 1.3 | 1.2 |
| MYST4 | 1.0 | 0.7 | 0.8 | 0.8 |
| N4BP2L1 | 1.1 | 0.9 | 0.9 | 0.8 |
| NAB1 | 1.0 | 0.9 | 0.7 | 0.9 |
| NCRNA00152 | 0.9 | 1.1 | 1.2 | 1.2 |
| NR1I2 | 0.7 | 0.9 | 0.8 | 0.8 |
| NRAP | 0.9 | 1.0 | 0.8 | 0.9 |
| NSUN3 | 1.1 | 1.2 | 1.3 | 1.2 |
| NTF3 | 0.9 | 2.0 | 2.2 | 1.6 |
| NTSR1 | 0.8 | 1.4 | 1.1 | 1.2 |
| NUDT12 | 0.9 | 0.9 | 0.6 | 1.2 |
| NUDT16 | 1.0 | 1.0 | 1.0 | 0.9 |
| NUDT8 | 1.6 | 1.4 | 1.4 | 1.4 |
| NXT1 | 0.9 | 1.1 | 1.1 | 1.1 |
| OAT | 0.8 | 0.8 | 1.0 | 0.9 |
| ODF3 | 1.1 | 1.2 | 1.3 | 1.0 |
| ODF3B | 0.9 | 0.8 | 0.8 | 0.8 |
| OLR1 | 1.2 | 1.1 | 1.4 | 1.0 |
| OR14J1 | 1.7 | 1.3 | 1.4 | 1.3 |
| OR2AK2 | 1.9 | 1.7 | 1.6 | 1.4 |
| OR4F21 | 0.5 | 0.6 | 0.7 | 0.5 |
| OR52D1 | 1.4 | 1.4 | 1.5 | 1.9 |
| OR7G2 | 1.3 | 0.9 | 1.4 | 1.2 |
| OR8K3 | 0.9 | 0.9 | 1.0 | 1.4 |
| OTC | 1.3 | 1.2 | 1.2 | 1.5 |
| OVGP1 | 1.4 | 1.3 | 0.9 | 1.0 |
| PAN3 | 1.2 | 1.0 | 0.9 | 1.1 |
| PCDHB4 | 0.9 | 0.8 | 1.0 | 1.0 |
| PCP2 | 1.0 | 1.1 | 0.7 | 0.6 |
| PDE10A | 0.8 | 0.9 | 1.1 | 0.8 |
| PECAM1 | 0.9 | 1.1 | 1.0 | 1.0 |
| PEPD | 1.0 | 0.9 | 0.9 | 0.9 |
| PEX1 | 0.9 | 0.8 | 0.6 | 0.9 |
| PFKFB2 | 1.0 | 0.8 | 0.8 | 0.9 |
| PFN3 | 1.5 | 1.3 | 1.2 | 1.4 |
| PHC1 | 0.9 | 0.9 | 0.8 | 0.9 |
| PIP5K2A | 0.9 | 1.0 | 1.2 | 1.1 |
| PLCB1 | 1.1 | 1.6 | 1.7 | 1.2 |
| PLCH1 | 0.9 | 0.5 | 0.7 | 0.8 |
| PPAN-P2RY11 | 1.0 | 0.8 | 0.7 | 0.9 |
| PPIAL4G | 1.1 | 1.2 | 1.5 | 1.2 |
| PRB3 | 1.2 | 1.2 | 1.3 | 1.0 |
| PRKAA2 | 0.7 | 1.0 | 1.1 | 0.9 |
| PRR3 | 1.1 | 0.9 | 1.1 | 1.2 |
| RAB33A | 1.2 | 1.4 | 0.9 | 1.2 |
| RARS | 0.9 | 0.9 | 1.0 | 1.0 |
| RBL2 | 1.1 | 0.9 | 0.8 | 0.9 |
| RGS2 | 1.3 | 1.1 | 0.5 | 0.6 |
| RGS5 | 1.3 | 1.0 | 1.2 | 1.0 |
| RNF152 | 0.8 | 0.7 | 1.0 | 0.9 |
| RPH3A | 1.1 | 1.4 | 1.5 | 1.2 |
| SBK2 | 0.9 | 1.0 | 0.6 | 0.7 |
| SDR9C7 | 1.1 | 1.2 | 1.1 | 1.1 |
| SHQ1 | 0.8 | 1.0 | 1.0 | 1.0 |
| SLC13A5 | 1.1 | 1.2 | 1.0 | 1.0 |
| SLC15A2 | 1.3 | 1.1 | 1.0 | 1.2 |
| SLC22A12 | 1.0 | 1.2 | 1.1 | 1.2 |
| SLC25A34 | 1.7 | 1.3 | 1.4 | 1.0 |
| SLC7A10 | 1.5 | 1.6 | 1.6 | 1.4 |
| SNORA15 | 0.8 | 0.8 | 0.8 | 1.0 |
| SNORD19 | 1.0 | 1.3 | 1.2 | 1.4 |
| SNX31 | 1.1 | 1.6 | 1.1 | 1.2 |
| SORCS1 | 0.8 | 0.9 | 0.8 | 1.0 |
| SORL1 | 1.0 | 1.0 | 0.8 | 0.9 |
| SPG20 | 1.1 | 0.9 | 0.8 | 0.7 |
| SRR | 0.9 | 0.9 | 0.9 | 0.8 |
| TAS2R38 | 0.5 | 0.7 | 0.8 | 1.0 |
| TCL6 | 0.9 | 0.9 | 0.8 | 0.9 |
| TDGF3 | 1.3 | 1.4 | 1.8 | 1.4 |
| TG | 0.6 | 1.0 | 1.0 | 0.9 |
| THBS3 | 1.1 | 0.9 | 0.6 | 0.8 |
| THRSP | 1.3 | 1.6 | 1.3 | 1.1 |
| THSD3 | 1.0 | 1.0 | 0.8 | 1.0 |
| TLR1 | 1.4 | 1.1 | 0.9 | 1.1 |
| TMSB4X | 0.8 | 1.2 | 1.5 | 1.1 |
| TMTC1 | 1.5 | 1.2 | 1.0 | 1.2 |
| TNFRSF8 | 1.5 | 1.2 | 1.2 | 1.2 |
| TOP1P1 | 0.9 | 0.8 | 0.8 | 0.9 |
| TRABD | 1.1 | 1.0 | 0.9 | 1.1 |
| TRAF3 | 1.0 | 0.7 | 1.1 | 1.1 |
| TREX2 | 0.8 | 0.7 | 0.8 | 0.6 |
| TRIM71 | 1.1 | 1.3 | 1.2 | 1.4 |
| TSTA3 | 0.8 | 1.0 | 1.2 | 1.1 |
| USP28 | 1.5 | 0.9 | 1.3 | 1.3 |
| VENTXP1 | 1.3 | 1.1 | 1.0 | 1.2 |
| WDR72 | 0.9 | 1.4 | 0.7 | 0.8 |
| WNK3 | 1.2 | 1.1 | 1.1 | 1.0 |
| XAGE2 | 1.1 | 1.2 | 1.1 | 1.1 |
| XIAP | 1.0 | 0.8 | 0.9 | 1.0 |
| YBX2 | 1.2 | 0.8 | 0.8 | 0.9 |
| ZAP70 | 0.9 | 0.9 | 1.1 | 0.9 |
| ZC3H11A | 1.0 | 1.3 | 1.0 | 0.8 |
| ZDHHC11 | 1.2 | 0.9 | 1.0 | 0.9 |
| ZIC1 | 1.0 | 1.4 | 1.2 | 1.3 |
| ZNF154 | 0.9 | 1.2 | 1.1 | 1.3 |
| ZNF281 | 1.2 | 1.0 | 1.0 | 0.9 |
| ZNF354A | 1.2 | 1.2 | 1.1 | 1.2 |
| ZNF367 | 0.8 | 1.5 | 1.1 | 1.4 |
| ZNF548 | 1.3 | 1.0 | 1.0 | 1.0 |
| ZNF596 | 1.1 | 0.9 | 0.8 | 0.8 |
| ZNF615 | 1.0 | 1.0 | 0.6 | 0.8 |
| ZNF644 | 1.0 | 0.9 | 0.9 | 1.0 |
| ZNF771 | 0.8 | 0.8 | 0.7 | 1.1 |
| ZNF808 | 1.0 | 0.7 | 0.8 | 1.0 |
| ZSCAN18 | 1.2 | 1.0 | 1.1 | 1.1 |

**Table S1.** **Differentially Expressed Genes**

Differentially expressed genes shown in Fig. 6a and their fold changes under different substrate stiffnesses.

| Abbreviation | **Full Information on Transcription Factors from the ChEA database** |
| --- | --- |
| HNF4A | HNF4A_19822575_ChIP-Seq_HepG2_Human |
| NUCKS1 | NUCKS1_24931609_ChIP-Seq_HEPATOCYTES_Mouse |
| WT1 | WT1_20215353_ChIP-ChIP_NEPHRON PROGENITOR_Mouse |
| FOXA2 | FOXA2_19822575_ChIP-Seq_HepG2_Human |
| DMRT1 | DMRT1_23473982_ChIP-Seq_TESTES_Mouse |
| YAP1 | YAP1_20516196_ChIP-Seq_MESC_Mouse |
| EGR1 | EGR1_20690147_ChIP-Seq_ERYTHROLEUKEMIA_Human |
| RELA | RELA_24523406_ChIP-Seq_FIBROSARCOMA_Human |
| TCF3 | TCF3_18467660_ChIP-ChIP_MESC_Mouse |
| ZFP281 | ZFP281_18757296_ChIP-ChIP_E14_Mouse |

**Table S2.** **Transcription Factors**

Full information associated with the network representation of transcription factors enriched from the protein-protein interaction network using the ChEA database shown in Fig. 6b in the main text.
